# Ferroptosis: Mechanism and connections with cutaneous diseases

**DOI:** 10.3389/fcell.2022.1079548

**Published:** 2023-01-04

**Authors:** Lihao Liu, Ni Lian, Liqing Shi, Zhimin Hao, Kun Chen

**Affiliations:** ^1^ Department of Physiotherapy, Institute of Dermatology, Chinese Academy of Medical Sciences, Peking Union Medical College, Nanjing, China; ^2^ Department of Dermatology, Institute of Dermatology, Chinese Academy of Medical Sciences, Peking Union Medical College, Nanjing, Jiangsu, China

**Keywords:** ferroptosis, programmed cell death, dermatology, cancer, inflammation

## Abstract

Ferroptosis is a recognized novel form of programmed cell death pathway, featuring abnormalities in iron metabolism, SystemXc^−^/glutathione axis, and lipid peroxidation regulation. A variety of ferroptosis inducers can influence glutathione peroxidase directly or indirectly *via* diverse pathways, leading to decreased antioxidant capacity, accumulated cellular lipid peroxides, and finally inducing ferroptosis. To date, mounting studies confirm the association of ferroptosis with various cutaneous diseases, including skin homeostasis, neoplastic diseases, infectious diseases, genetic skin disease, inflammatory skin diseases, and autoimmune diseases. There are shared characteristics regarding ferroptosis and various cutaneous diseases in terms of pathophysiological mechanisms, such as oxidative stress associated with iron metabolism disorder and accumulated lipid peroxides. Therefore, we summarize the current knowledge regarding the mechanisms involved in the regulation of ferroptosis for further discussion of its role in the pathogenesis and prognosis of skin diseases. Gaining insight into the underlying mechanisms of ferroptosis and the associated dermatological disorders could illuminate the pathogenesis and treatments of different cutaneous diseases.

## Introduction

In 2003, erastin, a novel compound was identified, which showed a selective lethality against human foreskin cells (BJeLR cells) expressing engineered mutated *RAS* oncogene while using high-throughput screening to find out 23,550 compounds for their ability to eliminate engineered tumorigenic cells (Dolma et al., 2003). But this pattern of cell death pathway was inconsistent with previous knowledge for there was no morphological alternation or DNA fragmentation. More importantly, this form of cell death cannot be reversed by caspase inducer [Boc-Asp (OMe)-fluoromethyl ketone] (Dolma et al., 2003). In 2008, Yang et al. found two brand new small molecules in a screening study that could specifically kill BJELR cells in a non-apoptotic way and named them Ras-selective lethal small molecule (RSL)3 and RSL5. RSL-induced cell death cannot be inhibited by apoptosis (z-VAD-fmk), necroptosis (necrostatin-1), and autophagy inhibitors (bafilomycin A1, 3-methyladenine, chloroquine) (Dixon et al., 2012; Yang and Stockwell, 2008). However, one iron chelator (deferoxamine mesylate) and one antioxidant agent (vitamin E) were capable of inhibiting cell death triggered by RSL, indicating the effects of iron as well as reactive oxygen species (ROS) (Cao and Dixon, 2016). Ferroptosis was eventually recognized as a novel, iron-dependent kind of programmed cell death (PCD) during the study of the mechanistic features of erastin-induced cell death by *RAS* mutations by Dixon et al. (2012).

### Mechanism of ferroptosis

**FIGURE 1:**
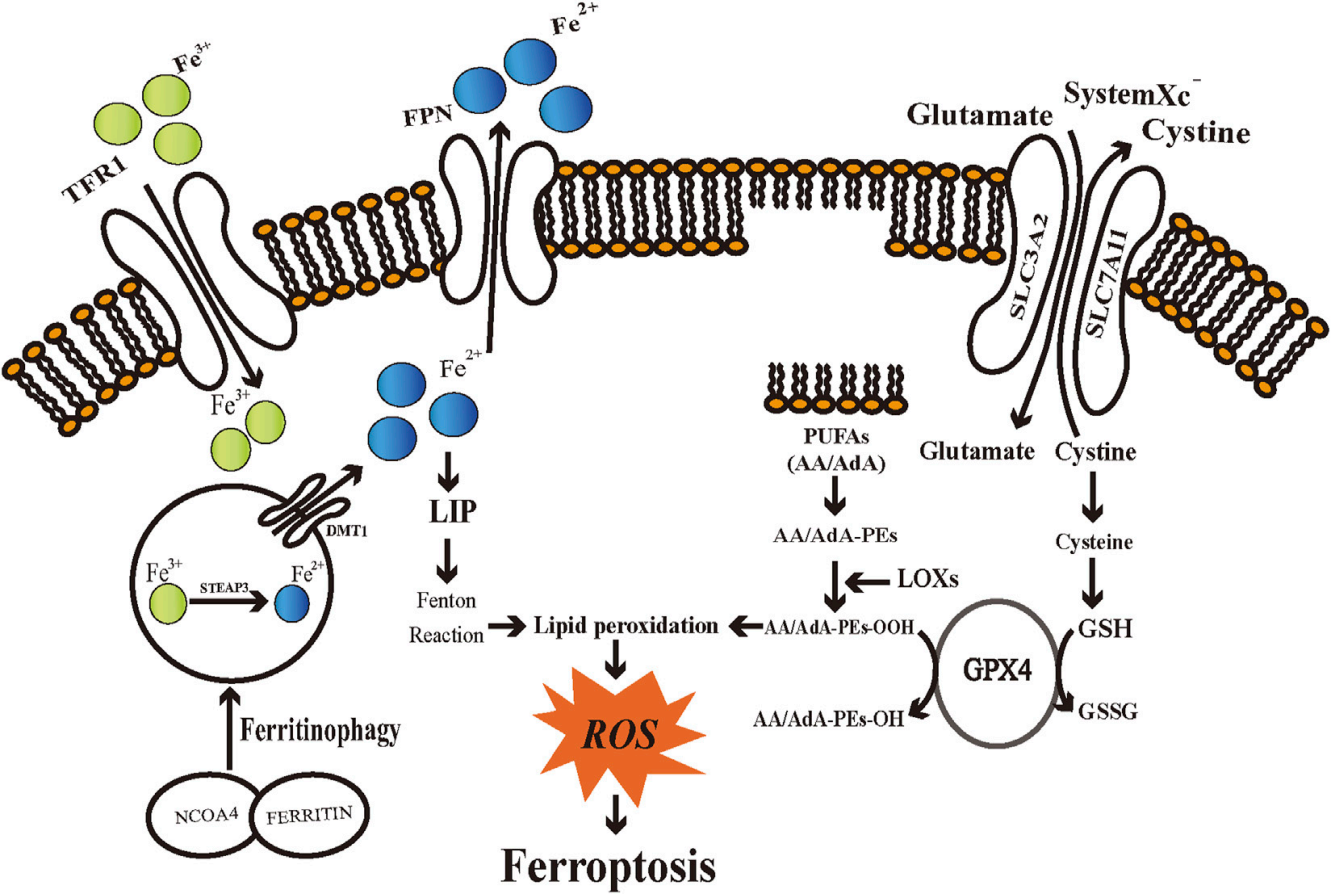
Schematic diagram of regulatory mechanisms of ferroptosis. Iron metabolic pathways, SystemX_c_^−^/glutathione axis, and lipid peroxidation engage in the regulation of ferroptosis. TFR1: transferrin receptor 1; FPN: ferroportin; DMT1: divalent metal transporter 1; STEAP3: six-transmembrane epithelial antigens of the prostate 3; NCOA4: Nuclear receptor coactivator 4; LIP: labile iron pool; ROS: reactive oxygen species; SLC3A2: Solute Carrier Family 3 Member 2; SLC7A11: Solute Carrier Family 7 Member 11; PUFAs: polyunsaturated fatty acids; AA/AdA: arachidonic acid/adrenic acid (AdA); AA/AdA-PEs: AA/AdA-phosphatidylethanolamine (PE); AA/AdA-PEs-OH: AA/AdA-PEs-alcohols; AA/AdA-PEs-OOH: AA/AdA-PEs-hydroperoxides; GPX4: Glutathione peroxidases 4; GSH: glutathione; GSSG: glutathione disulfide; LOXs: Lipoxygenases.

Ferroptosis has a distinctive morphological alteration compared with other PCDs such as apoptosis, necrosis, pyroptosis, and autophagy. The morphological alterations exhibited in ferroptotic cells include obviously smaller mitochondria, increased membrane density of mitochondria, reduced inner mitochondrial membrane folds, and loss of structural integrity (Dixon et al., 2012; Yagoda et al., 2007). Also, there was no typical morphological evidence of apoptosis, including swelling of the cytoplasm and organelles, nor rupture of the cell membrane (Dixon et al., 2012). Cells that have undergone ferroptosis remain at their normal nuclear size, without showing a lack of chromatin condensation, nuclear fragmentation, membrane blebbing, or formation of small vesicles known as apoptotic bodies, which are the signature morphological changes in apoptosis (Dixon et al., 2012; Xu et al., 2019). Such distinctive morphological alterations of ferroptosis help to distinguish it from other cell death pathways. Recent researches have illustrated cells undergo ferroptosis followed by changes in the metabolism and regulatory mechanisms. In this section, we present a summary of the mechanisms and critical regulators associated with ferroptosis from various aspects including iron metabolism, SystemX_c_^−^/glutathione axis, and lipid peroxidation regulation (Figure 1).

### Iron metabolism

Iron can generate ROS through enzymatic or non-enzymatic reactions. Therefore, iron has a significant role in enhancing cellular ferroptosis sensitivity (Feng and Stockwell, 2018). Intra- and extracellular iron remains in a dynamic balance. Disruption of such dynamic balance may influence the vulnerability of cells to ferroptosis. The divalent iron (Fe^2+^) generated through intestinal absorption or degradation by red blood cells is oxidized to trivalent iron (Fe^3+^), which becomes circulating iron by binding transferrin (TF) to form TF-Fe^3+^(Frazer and Anderson, 2014). By binding to transferrin receptor 1 (TFR1) and endocytosing the TF-TFR1 complex, imported iron then locates in the endosome (Yang and Stockwell, 2008). TFR1 recently has been introduced as a specific ferroptosis marker (Feng et al., 2020). Fe^3+^ is reduced to Fe^2+^ by the ferrireductase activity of six-transmembrane epithelial antigens of the prostate 3 (STEAP3) in the endosome (Knutson, 2007). Fe^2+^ is subsequently translocated into a labile iron pool (LIP) in the cytoplasm by divalent metal transporter 1 (DMT1, also known as Solute Carrier Family 11 Member 2, SLC11A2) (El et al., 2018). Ferritin, being a primary iron storage protein complex, can store excess intracellular iron by binding to chaperone proteins, known as Poly-(rC)-binding protein 1 (PCBP1) and PCBP2 (Ryu et al., 2017). The Intracellular ferrous iron can be oxidized and exported by the membrane protein ferroportin (FPN), which currently is the unique iron efflux pump discovered that can release iron from cells (Ganz, 2005).

The intracellular labile iron levels could be enhanced *via* TF-mediated iron absorption or selective autophagy of ferritin. It has been demonstrated that pseudolaric acid B could reinforce iron input by increasing TFR thereby triggering ferroptosis in glioma cells (Wang et al., 2018). In different tumor cell lines, the RAS–RAF–MEK pathway is capable of altering their susceptibility to ferroptosis (Dixon et al., 2012). In comparison to ferroptosis-resistant cells, cells sensitive to ferroptosis accompanied by RAS mutation exhibit elevated TFR1 expression and reduced ferritin expression, indicating that higher iron import and lower iron storage may lead to ferroptosis (Yang and Stockwell, 2008). One of the plausible explanations is that cancer cells with higher levels of iron can support fast proliferation (Liang et al., 2019). The lysosome is an essential site for cellular iron storage and is indispensable for maintaining a stable iron level. Autophagic degradation of ferritin in cytoplasm or lysosome, known as ferritinophagy, could lead to elevated intracellular iron levels, thereby accumulating ROS and eventually triggering ferroptotic cell death (Asano et al., 2011; Hou et al., 2016). Nuclear receptor coactivator 4 (NCOA4) is an autophagic cargo receptor of ferritin and is of importance to maintaining iron homeostasis (Mancias et al., 2014). Knockdown of NCOA4 inhibits ferroptosis in various tumor cell lines, which could be correlated with reduced intracellular free iron pools (Hou et al., 2016). This unique iron dependency renders tumor cells to become susceptible to iron overload, making it possible to develop ferroptosis-mediated therapy.

### SystemX_c_^−^/glutathione axis

SystemX_c_^−^ is a heterodimer cell surface amino acid transporter extensively located in the cell membrane, which is a vital component of the cellular antioxidant system (Dixon et al., 2012; Sato et al., 1999). SystemX_c_^−^ is composed of the transport subunit SLC7A11 and regulatory subunit SLC3A2 structurally, which are coupled by a disulfide bond (Sato et al., 1999). Back in 1980, studies indicated that SystemX_c_^−^ was capable of transferring cystine into cells and simultaneously exchanging glutamate at a ratio of 1:1 in an ATP-independent manner (Bannai and Kitamura, 1980; Sato et al., 1999). Cystine is the necessary precursor for synthesizing tri-peptide glutathione (GSH), which is a major endogenous antioxidant. GSH can be exhausted *via* inhibiting SLC7A11 by traditional drugs (e.g., sorafenib and sulfasalazine (SAS)) or novel compounds (e.g., erastin), thus inducing ferroptosis (Dixon et al., 2012,^2014^). Meanwhile, erastin initiates a feedback mechanism to regulate GSH overconsumption by upregulating SLC7A11 expression (Dixon et al., 2012). In addition, various factors engage in regulating the expression or activity of SLC7A11, such as BRCA1-associated protein 1 (BAP1) (Zhang et al., 2018), tumor protein 53 (TP53) (Jiang et al., 2015), nuclear factor E2 related factor 2 (NFE2L2/Nrf2) (Sun et al., 2016). In general, inhibiting the SLC7A11 pathway is a key mechanism for inducing ferroptotic cell death. Coenzyme A (CoA) can be synthesized through the pantothenate pathway with cysteine, and CoA inhibits SLC7A11 thereby causing ferroptosis (Badgley et al., 2020). It indicates that the non-GSH-dependent cysteine metabolism involves in the regulation of ferroptosis and also plays an important role in affecting the vulnerability of cells towards ferroptosis. GSH is synthesized from glutamate, cysteine, and glycine catalyzed by glutamate cysteine ligase (GCL) and glutathione synthetase (GSS) and involves in the regulation of ferroptosis (Kalinina and Gavriliuk, 2020; Lu, 2013). The most limiting material for synthesizing GSH is cysteine, and inhibiting its uptake through the SystemX_c_^−^ could induce ferroptosis *in vitro* (Doll and Conrad, 2017; Lu, 2013). Glutathione peroxidases 4 (GPX4) is a selenocysteine-containing, and GSH-dependent monomeric enzyme (Kelner and Montoya, 1998; Savaskan et al., 2007). In 2014, Yang et al. proposed that GPX4 serves as a critical regulator of ferroptosis in tumor cells through the inhibition of lipid peroxides formation. They revealed that cells with down-regulated GPX4 expression were more susceptible to ferroptosis, while the overexpression of GPX4 inhibits ferroptosis (Yang et al., 2014). This catalytic reaction of GPX4 follows a tert-uni ping pong mechanism, involving redox reactions of the selenocysteine (Ursini and Bindoli, 1987). The catalytic cycle involves three distinct basic reactions. First, the dissociated selenolate is oxidated by the peroxide substrate, generating a selenic acid derivative. Then, a GSH reacts with the oxidized enzyme and generates an intermolecular selenylsulfide bond. In the last step, a second GSH is used to regenerate the enzyme and form glutathione disulfide (GSSG) (Savaskan et al., 2007). Consequently, GSH is regarded as an essential factor in maintaining GPX4 activity. RSL3 inhibits the activity of GPX4, leading to a reduction in cellular antioxidant capacity and ROS accumulation, and ultimately to ferroptotic cell death (Yang et al., 2014). As mentioned above, selenocysteine is an important amino acid of the GPX4 active sites. The mevalonate (MVA) pathway exerts its effect on the GPX4 synthesis process by regulating selenocysteine tRNA, which is required to insert selenocysteine into GPX4. Consequently, suppressing the MVA pathway can downregulate GPX4 activity and induce ferroptosis (Warner et al., 2000). Furthermore, both cell development and the maintenance of different functions require the engagement of GPX4, as its genetic knockout or inactive form of expression leads to embryonic death lethality and subfertility in male mice (Cardoso et al., 2017; Ingold et al., 2015).

### Lipid peroxidation

Fatty acids serve as vital cellular nutrients and also maintain a wide range of cellular functions, including the formation of cellular membranes and the transmission of cellular signals (DeBose-Boyd,2018; Xie et al., 2020). Therefore, it is necessary to closely modulate fatty acid metabolism to avoid the toxicity noted in the cell death pathways, such as ferroptosis. Fatty acids in cells can derive from exogenous resources or *de novo* fatty acid synthesis (de Carvalho and Caramujo, 2018). There are a total of three kinds of different fatty acids, based on chemical bond differences, including saturated, monounsaturated, and polyunsaturated fatty acids (PUFA) -fatty acids with more than one double bond (de Carvalho and Caramujo, 2018). Studies show that PUFA-phospholipids (PUFA-PLs) are phospholipids that are most likely to undergo peroxidation reactions (Gaschler and Stockwell, 2017), especially phosphatidylethanolamine (PE)-containing phospholipids with arachidonic acid (AA) and adrenic acid (AdA). Bis-allylic carbons existing in such PUFA-PLs are prone to oxidation (Gill and Valivety, 1997). Acyl-CoA synthetase long-chain family member 4 (ACSL4) and lysophosphatidylcholine acyltransferase 3 (LPCAT3) serve important roles in integrating PUFAs into membranes (Dixon et al., 2015; Doll et al., 2017). The ligation reaction of CoA with AdA/AA is catalyzed by ACSL4 to form COA-AdA/AA intermediate, which subsequently undergoes esterification with lysophospholipids by LPCAT3. When PUFAs are bound to cell membranes, they can undergo peroxidation as well as ferroptosis. ACSL4 is a predictive biomarker of ferroptosis sensitivity, also a major contributor to ferroptosis (Doll et al., 2017; Yuan et al., 2016). However, ACSL4 is not necessary for all ferroptotic cell death, which means under special circumstances ferroptosis is able to be induced in ACSL4-depleted cells (Chu et al., 2019).

Ferroptosis is induced due to lipid peroxidation activated *via* non-enzyme-dependent (Fenton reaction) and enzyme-dependent processes. Iron exhibits redox activity and engages in the production of intracellular free radicals and lipid peroxides (Ayala et al., 2014). ROS are common intracellular oxidants, which can oxidate DNA, proteins, and lipids, thus disrupting biological homeostasis (Gaschler and Stockwell, 2017). ROS are generated from excessive intracellular iron *via* the Fenton reaction, which means reactions of Fe^2+^ and peroxides to generate oxygen-centered radicals (Winterbourn, 1995). The oxidation reaction between Fe^2+^ and H_2_O_2_ could generate hydroxyl radicals that yield hydrogen from PUFAs to form lipid radicals (L·). Lipid radicals bind with oxygen (O_2_) to produce a lipid peroxyl radical (LOO·), which yields hydrogen from adjacent PUFA to form lipid hydroperoxides (LOOH) and a lipid radical and simultaneously initiate a new lipid radical chain reaction (Hassannia et al., 2019).

Lipoxygenases (LOXs) are considered one of the central players in ferroptosis (Ayala et al., 2014). Enzymatical lipid peroxidation is regulated by LOXs, and suppression or downregulation of LOXs activity leads to inhibition of ferroptosis in certain cell lines (Shah et al., 2018). LOXs, a group of iron-containing enzymes, are responsible for catalyzing the PUFAs (with a 1,4-*cis, cis* pentadiene system) oxidation through stereo-specific peroxidation to produce corresponding fatty acid hydroperoxides (Kuhn et al., 2018). Based on expression differences in individual tissues, there are six arachidonate lipoxygenase (*ALOX*) genes in humans (Mashima and Okuyama, 2015). The *ALOX* family can mediate PUFAs peroxidation to generate AA/AdA-PE-OOHs, thereby triggering ferroptosis (Lei et al., 2019). According to the specific oxidation position, the 12/15-LOX is in charge of catalyzing AA to 12-/15- hydroperoxyeicosatetraenoic acid (12-/15-HpETE) (Ackermann et al., 2017). Chu et al. (2019) discovered that the inactivation of 12-LOX inhibits TP53-mediated ferroptosis and abrogates TP53-dependent inhibition of tumor growth, indicating that 12-LOX is vital for TP53-dependent cancer suppression. 15-LOX and 15-LOX-B undergo localization to biological membranes and selectively oxygenate PE-AA thereby inducing ferroptosis (Snodgrass and Brune, 2019). Furthermore, sn2-15-hydroperoxy-eicasotetraenoyl-phosphatidylethanolamines (sn2-15-HpETE-PE) generated by 15-LOX phosphatidylethanolamine binding protein-1 (PEBP1) complex can serve as a marker for ferroptosis (Anthonymuthu et al., 2018). 12/15-LOX shares a large substrate scope, such as linoleic acid and docosahexaenoic acid (Dobrian et al., 2011). Nonetheless, the controversy over LOXs central regulatory role in ferroptosis remains. It has been found that 12/15-LOX inactivation was unable to rescue the death of embryos in GPX4 knockout mice or to prevent cell death in GPX4 knockout mice (Brutsch et al., 2015; Friedmann et al., 2014). Certain cell lines showed susceptibility to ferroptosis but without expressing any detectable amounts of major LOX enzyme (Shah et al., 2018). Consequently, what part LOX plays in ferroptosis is open to question and requires further investigation.

As mentioned above, membranes can be protected from peroxidation damage *via* a GSH-dependent lipid peroxide repair system. GPX4 is able to directly reduce toxic LOOH into innocuous lipid alcohols by the action of GSH (Deponte, 2013). Cell death would be induced when regulators of lipid peroxidation malfunction in the chain reactions catalyzed by iron and ROS. Lipid peroxidation in cellular membranes changes the intrinsic characteristics of phospholipid membranes, including disrupting ion gradient, reducing membrane fluidity, and increasing membrane permeability (Catala and Diaz, 2016; Wong-Ekkabut et al., 2007). It has been suggested that protein-based pores contribute to ferroptosis, thus leading to ion gradient disruption (Magtanong et al., 2016). Molecular dynamic investigations proposed that, in the process of ferroptosis, alterations in lipid composition cause changes in cellular membrane shape and curvature, leading to exposure to more oxidants, irreversible destruction to the membrane integrity, and ferroptotic cell death (Agmon et al., 2018). Lipid peroxides of PUFAs may generate various toxic oxidation derivatives, including LOOHs and aldehydes. 4-hydroxynonenal (4-HNEs) and malondialdehyde (MDA) are the most abundant toxic byproducts that react with DNA bases or other vital proteins, resulting in serious cytotoxicity and further promoting ferroptosis (Ayala et al., 2014). Not only regarded as a critical biomarker for lipid peroxidation, but 4-HNE also involves regulating Nrf2 and Nuclear factor-kappa B (NF-κB) pathway (Jaganjac et al., 2020). Meanwhile, 4-HNE influences cell the process of cell development and differentiation and closely associates with other PCDs patterns, including autophagy, apoptosis, and necrosis (Ayala et al., 2014).

### Inducer

There are currently four types of ferroptosis inducers. The first class of inducers acts by preventing cysteine import (Hirschhorn and Stockwell, 2019). Such compounds are able to inhibit SystemX_c_^−^, thus leading to exhaustion of cysteine in cells, GPX4 inactivation, accumulated lipid peroxides, and eventually ferroptotic cell death. Erastin is one of the representative agents of the first class and the prototype ferroptosis inducer that depletes GSH by irreversibly binding to SLC7A11, thus inactivating it (Dolma et al., 2003). Voltage-dependent anion channels (VDACs) are mitochondrial transmembrane channels and are responsible for transporting ions and metabolites (Fang and Maldonado, 2018). The research team identified that erastin could directly bind to VDAC2/3, leading to loss of mitochondrial function as well as the emission of substantial amounts of oxides, finally causing ferroptosis (Yagoda et al., 2007). Furthermore, Wu et al. demonstrated that erastin-induced ferroptosis elevated lysosome-associated membrane protein 2a (LAMP2a) expression and promoted chaperone-mediated autophagy (CMA) while facilitating GPX4 degradation, suggesting a possible connection between autophagy and ferroptosis (Wu et al., 2019). SAS is used to treat chronic inflammation in joints (Ward et al., 2019), inflammatory bowel disease (Jeong et al., 2019), and various cutaneous diseases, including alopecia areata, lichen planus, and chronic idiopathic urticaria (Cranwell et al., 2019; Orden et al., 2014; Puza and Cardones, 2017). SAS is able to inhibit SystemX_c_^−^ thereby inducing ferroptosis in addition to inhibition of NF-κB pathway (Gout et al., 2001). However, whether SAS serves as an inducer of ferroptosis during treatments of cutaneous diseases remains unknown. The second class of inducers can directly inhibit GPX4 and cause ferroptosis. (*1S,3R*)-RSL3 forms covalent bonds with GPX4, which is inactivated *via* alkylating the selenocysteine and leads to toxic lipid peroxides buildup, and inevitable cell death (Yang et al., 2014; Yang and Stockwell 2008). There are four kinds of stereoisomers of RSL3, however, only (*1S,3R*)-RSL3 exhibits a lethal effect on four genetically modified HRAS^V12^-harbouring cell lines (Yang et al., 2014). The third category includes ferroptosis inducer 56 (FIN56) and caspase-independent lethal 56 (CIL56). FIN56 is one ferroptosis inducer derived from CIL56, which is capable to induce cell death without activating caspases 3/7 (Shimada et al., 2016). Studies showed that the lethality of low levels of CIL56 could be effectively prevented by antioxidants and iron chelators. However, researchers suggested that in the case of high concentrations of CIL56, which induces non-suppressible necrotic death (Shimada et al., 2016). FIN56 can act by depleting GPX4. In addition, FIN56 is capable of binding and activating squalene synthase, and leads to depleting coenzyme Q10 (CoQ10) (Shimada et al., 2016), which is a vital antioxidant by directly reducing lipid peroxyl radicals (Ernster and Forsmark-Andree,1993). The final category includes FINO_2_, which is able to oxidate iron and indirectly inactivate GPX4, thus inducing lipid peroxidation in an ALOX-independent manner (Abrams et al., 2016; Gaschler et al., 2018).

## Connection

Although the majority of current research regarding ferroptosis has focused on malignancies and degenerative diseases, there remain a few investigations on ferroptosis in cutaneous diseases. The etiology of various cutaneous diseases, such as malignant tumors or photoaging, is tightly associated with prolonged exposure to pro-oxidant environmental elements, including ultraviolet and chemical oxidants. In the skin, ROS are generated continuously, however, they can be eliminated by a variety of antioxidant substrates (Richelle et al., 2006). In case of depletion of the intracellular antioxidant system, there will be cellular damage. Cell death relates to a number of cellular heterogeneous processes. The organic peroxide *tertiary*-butyl hydroperoxide (t-BuOOH) is one of the most commonly employed agents to generate ROS in experiments (Xiao et al., 2005). The t-BuOOH-induced human keratinocyte death could be suppressed by ferroptosis blockers (ferrostatin-1, liproxstatin-1), lipid antioxidant α-tocopherol, and the iron chelator deferoxamine (Wenz et al., 2018). Sengupta et al. (2010) demonstrated that selenoproteins are indispensable in skin development by knocking out the selenocysteine tRNA gene in keratin14-expressing cells, which generated mice with epidermal hyperplasia and abnormal hair follicle development. This investigation also revealed GPX4 gene expression level is one of the higher expressed selenoprotein genes in the epidermis of mouse skin and cultured keratinocytes. Later, the same research team found that keratinocyte-specific knockout mice lacking GPX4 showed aberrant epidermis proliferation, infiltration of inflammatory cells in the dermis, and morphologic alteration of hair follicles. GPX4 knockdown keratinocytes appeared to have a decreased capacity for cell adhesion, along with accumulating lipid peroxides and enhanced levels of various pro-inflammatory elements, including COX-2, 4-HNE, and prostaglandins (Sengupta et al., 2013). Therefore, we cannot overlook how ferroptosis contributes to the development of the skin and its appendages and the pathophysiological processes of different skin diseases.

## Skin homeostasis

**FIGURE 2:**
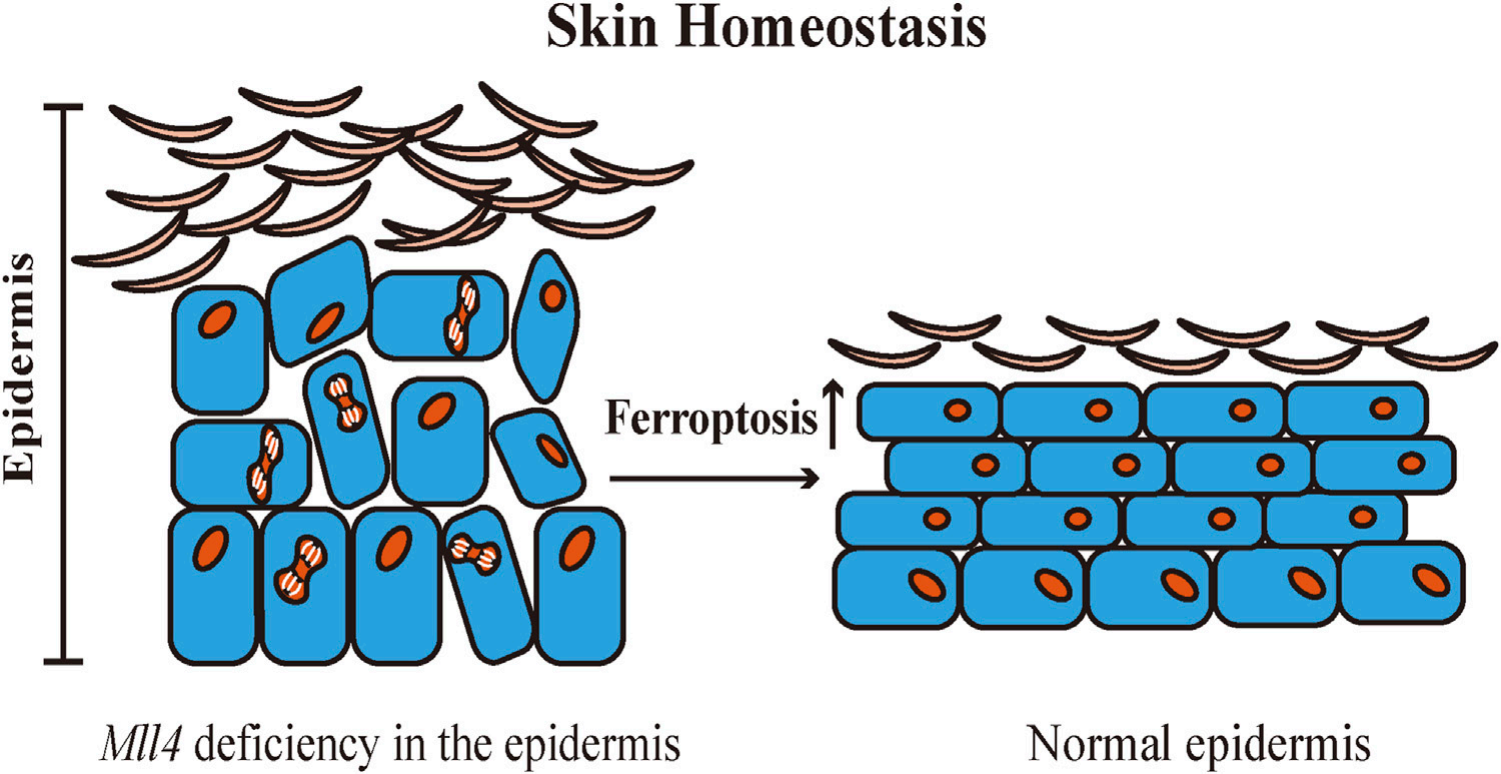
Ferroptosis contributes to the regulation of normal epidermal differentiation. Ferroptosis deficiency may induce abnormal differentiation of the epidermis and disrupt skin homeostasis. Mll4: Histone-lysine N-methyltransferase 2D (KMT2D, MLL4).

Skin is one of the most significant protective organs of the body. It defends against exogenous and endogenous stressors, prevents loss of water and nutrients, and maintains skin homeostasis (Passeron et al., 2021). The structural components of the skin barrier include keratinocytes, internal structural proteins, and specific skin lipids (Cui et al., 2016). Abnormalities in the structural components can disrupt the skin barrier and destabilize skin homeostasis, causing various cutaneous diseases (Yosipovitch et al., 2019). Histone-lysine N-methyltransferase 2D (KMT2D, MLL4) is an important epigenetic regulator (Froimchuk et al., 2017). Egolf et al. (2021) created *Mll4* epidermal-specific knockout mice and found that Mll4 deficiency in the epidermis impacts epidermal differentiation and disrupts skin homeostasis in mice. Transcriptome sequencing revealed that *Mll4* deficiency in the epidermis was correlated with the downregulation of multiple *Alox* genes expression. Moreover, it was confirmed that ferroptosis is involved in promoting normal epidermal differentiation, and epidermal barrier formation and serves to inhibit skin squamous cell carcinoma. A substantial amount of diverse LOX metabolism is found in the mammalian epidermis. LOX also oxidizes ceramides, which are essential in maintaining skin barrier function (Krieg and Furstenberger, 2014). 15-LOX and its oxygenation products affect cellular function by modifying biomembrane structure, and 15-LOX has a dual effect on the inflammatory response. The 15-LOX metabolic pathway promotes the synthesis of anti-inflammatory mediators to induce anti-inflammatory processes (Colakoglu et al., 2018). Kim et al. (2018) identified the anti-inflammatory mechanism of ALOX15 in skin homeostasis. The infiltration of macrophages in the dermal adipose tissue of *Alox15* knockout mice induced necroptosis in the dermis and dermal adipose tissue, and chronic inflammation in both the dermis and epidermis, resulting in disruption of the skin barrier. In contact dermatitis and atopic dermatitis, inhibition of 15-LOX inhibits podosome formation in dendritic cells, downregulates the antigen uptake and cell migration capabilities, and contributes to the alleviation of skin inflammation (Han et al., 2017). In the pathway of ferroptosis, 15-LOX is a prototypical enzyme in the process of lipid peroxidation. Also, 15-LOX is a key metabolic enzyme in maintaining skin homeostasis. Nevertheless, whether 15-LOX participates in the maintenance of skin homeostasis by triggering ferroptosis through lipid peroxidation remains to be further explored (Figure 2).

## Neoplastic disease

**FIGURE 3:**
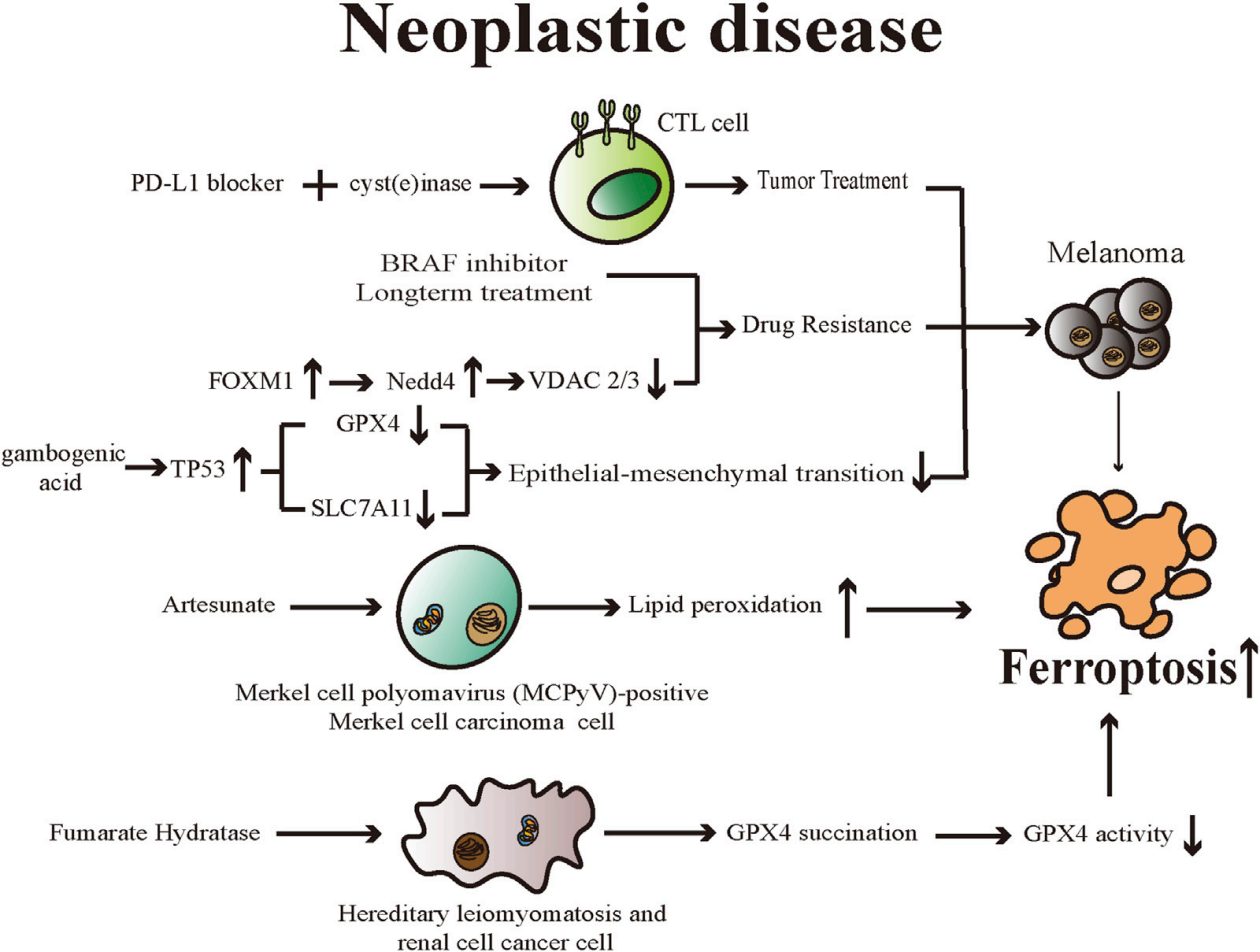
Various drugs promote ferroptosis in diverse neoplastic diseases through different mechanisms. Ferroptosis is emerging as a promising therapeutic target for neoplastic diseases. CTL cell: cytotoxic T lymphocytes; BRAF: v-RAF murine sarcoma viral oncogene homolog B1; FOXM1: Forkhead Box M1; Nedd4: neuronal precursor cell-expressed developmentally downregulated 4; VDAC: Voltage-dependent anion channels; TP53: tumor protein 53; GPX4: Glutathione peroxidases 4; SLC7A11: Solute Carrier Family 7 Member 11.

Cell death is vital for the body in maintaining normal development and homeostasis as well as preventing diseases such as tumors. Despite numerous breakthroughs that have been made in cancer treatments, however, the emergence of non-response to chemotherapy because of genetic mutations is an issue that cannot be overlooked (Lu et al., 2017). Ferroptosis is related to various pathophysiological processes in the body, particularly therapeutic options for many kinds of neoplastic disease. Different researchers have established the importance of ferroptosis in suppressing tumor cell proliferation and eliminating them. Hence, it is possible that exploiting ferroptosis-related therapeutic regimens may be a novel measure for treating tumors (Figure 3).

### Tumor treatment

Wang et al. found that a programmed cell death ligand 1 (PD-L1) inhibitor reduced tumor growth and increased oxidized lipids in cancer cells when studying mice with melanomas, suggesting that PD-L1 inhibitor induces ferroptosis (Wang et al., 2019). The research team confirmed that interferon-γ (IFNγ) generated by tumor-infiltrating cytotoxic T lymphocytes (CTL) can mediate anticancer effects through ferroptosis. And they also proved IFNγ downregulated the mRNA expression levels of both SystemX_c_^−^ subunits, leading to decreased GSH levels and inducing ferroptosis in tumor cells. In mouse models, the team introduced combined therapy including PD-L1 blocker and cyst(e) inase (a bioengineered enzyme that degrades cystine and cysteine) and such treatment can enhance the efficacy of CTL-mediated anticancer effects, supporting the idea that inducing ferroptosis could be considered a productive way for treating cancer and overcoming immunotherapy resistance (Wang et al., 2019).

Merkel cell carcinoma (MCC) is a rare, malignant neuroendocrine cutaneous tumor (Tello et al., 2018). Artesunate can be used to treat malaria and is derived from artemisinin, which is one of the main components of the herbal medicine *Artemisia annua* (Mancuso et al., 2021). Sarma et al. (2020) discovered artesunate inhibited the growth of cultured Merkel cell polyomavirus (MCPyV)-positive MCC cell lines effectively, and artesunate-induced cell death in investigated MCC cell lines can be significantly reduced by ferrostatin-1, deferoxamine and rosiglitazone (an inhibitor of the ACSL4). The experiments indicated that MCPyV-positive MCC cells could be eliminated by artesunate through dysregulation of lipid peroxides and eventually triggering a ferroptotic process. In addition, artesunate can effectively reduce tumor growth of MCPyV-positive tumors in xenotransplantation mouse models, further confirming the ability of artesunate to suppress the proliferation of MCPyV-positive MCC cells and the viability of combination therapy with other therapeutic options for MCC (Sarma et al., 2020).

### Drug resistance

Cell dedifferentiation is one of the signs of tumor progression and promotes resistance to current therapies (Gupta et al., 2019). Tsoi et al. (2018) found melanoma cells could be induced to dedifferentiated by supplying IFN-γ and tumor necrosis factor-α(TNF-α) to melanoma culture to mimic the inflammatory tumor microenvironment and such microenvironment is associated with increased susceptibility to ferroptosis induction. In addition, the team demonstrated an inverse relationship between the susceptibility of melanoma cells to ferroptosis and their differentiation degree using a pharmacogenomics integration analysis. The emergence of resistance to long-term treatment of melanoma with v-RAF murine sarcoma viral oncogene homolog B1 (BRAF) inhibitors and the related cellular dedifferentiation enhances the vulnerability of cells to various ferroptosis inducers. These results confirm the ability and value of triggering ferroptosis, thereby overcoming innate or dedifferentiation-associated resistance due to traditional therapies (Tsoi et al., 2018).

As mentioned above, erastin can directly bind to VDAC2/3 and cause ferroptosis. Meanwhile, following a prolonged period of erastin treatment, VDAC2/3 failed to be detected in the cells and such reduced expression resulted in erastin resistance (Yagoda et al., 2007). Yang et al. illustrated that neuronal precursor cell-expressed developmentally downregulated 4 (Nedd4), is capable of ubiquitylating as well as degrading VDAC2/3, thereby modulating ferroptotic processes in melanoma cells triggered by erastin specifically (Yang et al., 2020). The researchers found that the elevated ROS level triggered by erastin can stimulate Nedd4 expression by inducing Forkhead Box M1 (FOXM1), an essential regulator for oxidative stress and cancer cell survival. Depleting Nedd4 can reduce the degradation of VDAC2/3, thus increasing the vulnerability of tumor cells to erastin (Yang et al., 2020). Such a negative feedback loop offers a resolution for overcoming ferroptosis inducer resistance.

### Epithelial-mesenchymal transition

Epithelial-mesenchymal transition (EMT) is a crucial mechanism closely associated with the ability of cancer cells to invade and metastasize distantly (Nieto et al., 2016). Through this process, tumor cells exhibit higher migratory and invasive ability, poorer outcomes, and increased resistance to various treatments (Li et al., 2015). Wang et al. (2020) revealed that gambogenic acid (GNA) could suppress the invasive and migratory capacities as well as the EMT process in melanoma cells, which showed signature ferroptosis morphological changes. Through upregulating TP53 activity, GNA lowered the expression levels of GPX4 and SLC7A11, leading to dysregulation of cellular lipid peroxides, and eventually ferroptosis in transforming growth factor-β1 (TGF-β1)-induced EMT melanoma models (Wang et al., 2020).

### Gene mutation

It is hypothesized that triggering ferroptosis might be available for the targeted treatment of tumors with RAS oncogenic mutations (Dixon et al., 2012). Both RAS and RAS-independent pathways can induce ferroptotic cell death (Yu et al., 2015). Hereditary leiomyomatosis and renal cell cancer (HLRCC) is a syndrome of inherited cancers, including cutaneous leiomyomas (CLMs), uterine fibroids, and aggressive kidney tumors (Tomlinson et al., 2002). HLRCC syndrome is caused by an autosomal dominantly inherited mutation of the fumarate hydratase (*FH*) gene (Tomlinson et al., 2002). Reduced FH enzyme activity leads to the accumulation of the cellular level of fumarate, and fumarate can undergo a reaction with cysteine residues of proteins, which is called succination (Blatnik et al., 2008; Pollard et al., 2005). A study showed *FH*-knockout (*FH* ^*−/-*^) cells are sensitive to ferroptosis, and such sensitivity is attributed to dysfunctional GPX4 (Kerins et al., 2018). Mechanically, due to the accumulated fumarates, C93 of GPX4 undergoes post-translational succination modification in *FH* ^*−/-*^ cells, and the reaction reduces the activity of GPX4 (Kerins et al., 2018). Such sensitivity to ferroptosis can be considered the foundation of pharmaceutical development for killing HLRCC cells selectively in the future.

## Infectious disease

The skin is in some sense a defense organ representing an essential barrier against external microbial insults. Cell death is an important issue in infectious diseases. There are pieces of evidence suggesting ferroptosis contributes to the pathophysiological process of multiple infectious microorganisms. In mice with T cell-specific deletion of *Gpx4*, microbes-specific CD8^+^ and CD4^+^ T cells were unable to proliferate normally meanwhile exhibiting enhanced sensitivity towards viral and parasitic infections. CD8^+^ T cells of the knockout mice were intrinsically defective in balancing homeostasis in the peripheral environment, implicating GPX4 is required for T cell-regulated immune responses (Matsushita et al., 2015). Human immunodeficiency virus (HIV)-infected cell death could be triggered by a variety of PCD pathways, including apoptosis, pyroptosis, and ferroptosis (Cao et al., 2020). Specifically, by establishing a CD4^+^T cell line for infecting HIV, Cao et al. (2020) demonstrated HIV promoted CD4^+^T cell death through apoptosis and pyroptosis in CD4^+^T cells (especially in p24^+^ cells) and ferroptotic cell death (especially in p24^-^ cells) in the initial phase of infection. CD4^+^T cell death was related to abnormal DNA and telomere damage and the emergence of diverse cell death pathways were associated with the expression of pro-/anti-apoptosis proteins and signaling molecules (Cao et al., 2020).

**FIGURE 4:**
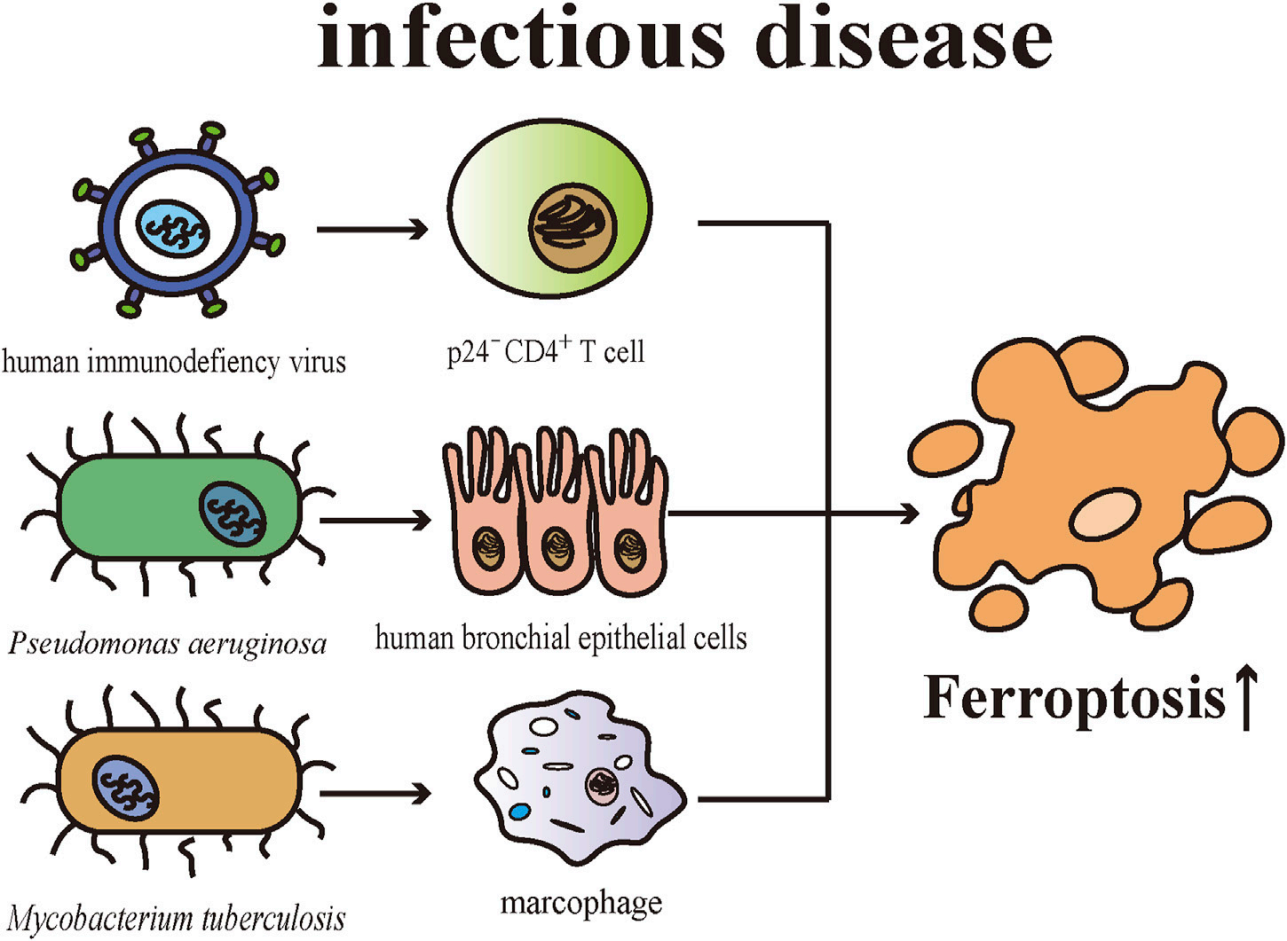
Various pathogenic microorganisms engage in the pathogenesis of infectious diseases by inducing ferroptosis in different cells.

Cellular membranes have a role in defending against infection and maintaining the homeostasis of host cells. Studies have found that lipid peroxidation is associated with pathophysiological processes of bacterial and viral infections (Martynenko et al., 1990; Toufekoula et al., 2013). PUFAs being esterified into membrane phospholipids shows a vulnerability to the invasion of microbial (Mao et al., 2020). There are mounting shreds of evidence proving that *Pseudomonas aeruginosa* (*P. aeruginosa*) causes damage to the epithelium, while simultaneously impairing the epithelial repair mechanism after injury (Ruffin and Brochiero, 2019). *P. aeruginosa* can induce ferroptotic cell death in human bronchial epithelial cells by expressing lipoxygenase (pLoxA), which can oxidize host PE-AA to 15-HpETE (Dar et al., 2018). *P. aeruginosa* obtained from patients suffering from chronic respiratory tract infections can induce ferroptosis and such cell death is closely related to the expression level and catalytic efficiency of pLoxA (Dar et al., 2018). Iron generates ROS by catalytic reactions to remove microbial stimulation as a part of the innate antimicrobial defense (Mao et al., 2020). *Mycobacterium tuberculosis (M. tuberculosis)*-induced macrophage necrosis showed elevated cellular iron levels, mitochondrial superoxide and lipid peroxides, and reduced levels of GPX4 and GSH, which are essential features of ferroptosis (Amaral et al., 2019). Furthermore, the death of *M. tuberculosis*-induced macrophage can be inhibited by both ferrostatin-1 and iron chelators (Amaral et al., 2019). Consistent with expectations, ferrostatin-1-treated mice exhibits reduced levels of lipid peroxidation, and the severity of tissue necrosis caused by *M. tuberculosis* as well as bacterial load are limited through inhibiting ferroptosis with ferrostatin-1 (Amaral et al., 2019). Both *P. aeruginosa* and *M. tuberculosis* infections are common opportunistic pathogens and can affect the skin causing corresponding skin lesions. Globally, both share the challenge of severe antibiotic resistance in their individual treatments (Ibrahim et al., 2020; Koch and Mizrahi, 2018). Despite the fact that the ferroptosis triggered by these pathogens has not been validated in skin tissues, these studies bring new insights into the research of their respective pathogenesis and therapeutic targets (Figure 4).

## Genetic skin disease

**FIGURE 5:**
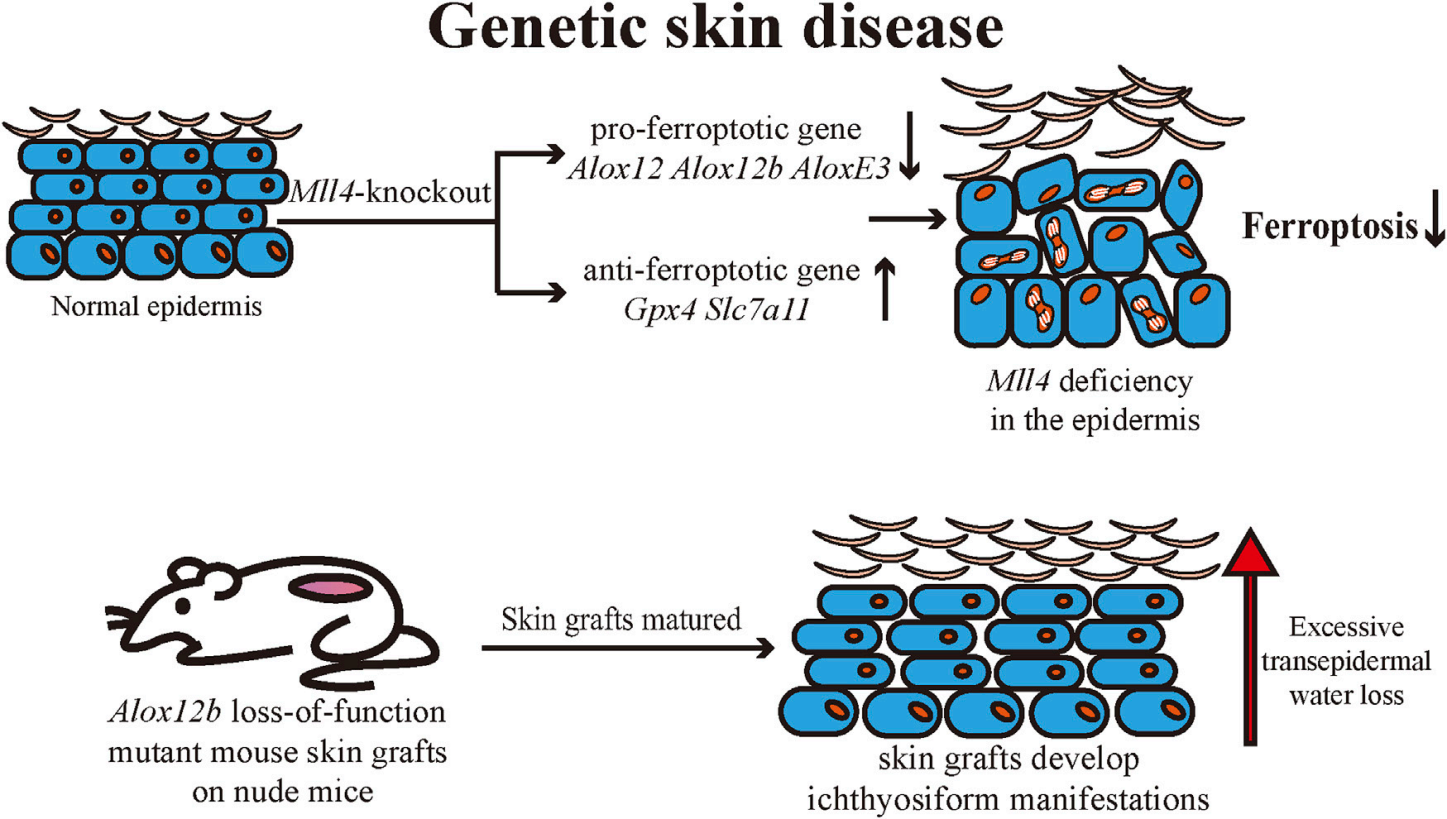
*Mll4*-specific deficiency in mouse epidermis leads to abnormal expression of several ferroptosis-related genes in keratinocytes, resulting in reduced ferroptotic cell death and consequently affecting normal epidermal differentiation and disrupting the skin barrier. The pathogenesis of autosomal recessive congenital ichthyosis is associated with multiple ALOX genes, and it is hypothesized that ferroptosis may be involved in the pathogenesis of ichthyosis. Mll4: Histone-lysine N-methyltransferase 2D (KMT2D, MLL4); ALOX: arachidonate lipoxygenase; SLC7A11: Solute Carrier Family 7 Member 11; GPX4: Glutathione peroxidases 4.

Ichthyosis is a group of genetic dermatoses with common clinical manifestations including generalized dry skin, erythema, desquamation, hyperkeratosis, and skin inflammation (Takeichi and Akiyama, 2016). Ichthyosis is usually caused by genetic mutations that affect various cellular functions, including DNA repair, lipid synthesis, and adhesions, resulting in skin barrier dysfunction (Limmer et al., 2020). It has been established that ALOX12, ALOX12B, and ALOXE3 mutations can cause autosomal recessive congenital ichthyosis (ARCI) (Mashima and Okuyama, 2015). Statistics show that LOX gene mutations are the second most common cause of ARCI (Krieg and Furstenberger, 2014). 12R-LOX and eLOX-3 are engaged in the synthesis of the cornified envelope (CE) through the enzymatic synthesis of lipid oxide products in the epidermis to maintain the skin barrier and reduce transepidermal water loss (TEWL) (Krieg and Furstenberger, 2014). Ethylnitrosourea induces a loss-of-function mutation in mice in *Alox12b*. Mutant mice were born with red, shiny skin that rapidly dried and died, lacking typical ichthyosiform lesions (Moran et al., 2007). *Alox12b* loss-of-function mutant mouse skin grafts can develop local ichthyosiform manifestations when matured on nude mice. Histologically, epidermal thickening, severe hyperkeratosis, and epidermal acanthosis were seen (de Juanes et al., 2009). Egolf et al. found that epidermis-specific *Mll4*-knockout mice developed red, scaly skin (Egolf et al., 2021). Transcriptomic analysis of the epidermis revealed that *Alox12*, *Alox12b*, and *AloxE3* expressions were downregulated. The research team confirmed that *Alox12* expression in the epidermis was regulated by the epigenetic regulatory function of MLL4. The expression of pro-ferroptosis genes was also found to be downregulated in the epidermis of mutant mice, and the expression of ferroptosis-suppressing genes such as *Gpx4* and *Slc7a11* was upregulated. The role of ferroptosis in regulating normal epidermal differentiation was hypothesized and confirmed by treating an iron chelator to human skin organoids (Egolf et al., 2021). Both epidermis-specific *Mll4-*knockout mice and *Alox12b* loss-of-function mutant mice exhibited epidermal hyperproliferation, scaling, and other cutaneous manifestations similar to ichthyosis, as well as a deficiency of ALOX12b expression in the epidermis of both mice. Accordingly, we can hypothesize that ferroptosis may serve to regulate normal epidermal differentiation during the pathophysiology of ichthyosis. Currently, the relationship between ichthyosis and ferroptosis remains to be further explored (Figure 5).

## Inflammatory skin disease

As a common, immune-related inflammatory cutaneous disease, psoriasis affects not only the skin but also multiple systems (Boehncke and Schon, 2015). Recognizing psoriasis as a systemic inflammatory disease is beneficial for disease management and optimizing patient prognosis (Reich, 2012). ROS and oxidative stress are common features associated with inflammation (Mittal et al., 2014). Oxidative stress is well-established to be closely related to the pathogenesis of psoriasis (Kirmit et al., 2020). The external adverse elements, such as cigarette smoking, physical damage, and microbiota influence, may cause keratinocytes damaged through excessive production of ROS(Plenkowska et al., 2020). Even though the immune system responds towards inflammatory stimuli through ROS, increased ROS levels can damage DNA, proteins, and lipids, and result in activating various signaling pathways (such as NF-κB and mitogen-activated protein kinase (MAPK)), stimulating T helper cells 1 (Th1) and Th17 cells, and promoting the release of inflammatory cytokines (Plenkowska et al., 2020), which can contribute to the development and exacerbation of the psoriatic inflammation.

There are differences found in bioactive lipid mediators of skin and blood samples between psoriatic patients and healthy individuals using liquid chromatography-tandem mass spectrometry approaches (Sorokin et al., 2018). AA metabolites were remarkably elevated in psoriatic lesions compared to healthy individuals and non-lesioned skin samples. Free 15-HpETE elevated noticeably in psoriatic lesions compared to healthy individuals (Sorokin et al., 2018). An evaluation investigating the relationship between elevated oxidative stress markers and psoriasis severity found that the most commonly elevated marker is MDA, which is correlated with psoriasis severity and associated with ferroptosis (Ayala et al., 2014; Cannavo et al., 2019). The majority of the oxidative stress markers are generated during lipid metabolism and might be associated with aberrant metabolism in psoriasis (Cannavo et al., 2019). A study suggests that abnormal phospholipids metabolism, notably PUFAs, is the central element in the pathogenesis of oxidative stress in psoriasis (Ambrozewicz et al., 2018). Single-cell RNA sequencing analysis revealed that the lipid oxidation activity of keratinocytes was closely related to the Th22/Th17 pathway, and lipid peroxidation in keratinocytes was also found to be enhanced during the course of psoriasis (Shou et al., 2021). During lipid peroxidation, cyclic guanosine monophosphate (cGMP) is elevated, along with the downregulation of cyclic adenosine monophosphate (cAMP) concentration, leading to accelerated proliferation of epidermal cells in psoriasis (Aksoy and Kirmit, 2020).

Extensive experiments are proving that various subsets of T cells, including Th1, Th2, Th17, and regulatory T cells (Treg cell), as well as related cytokines, such as TNF-α, IFN-γ, IL-23, and IL-17, engage in the pathogenesis of psoriasis (Deng et al., 2016). Studies have pointed out that Treg cells have an indispensable part in the development of psoriasis (Hartwig et al., 2018). The impaired Treg cell function, phenotypic alteration and disrupted Treg cell/Th17 cell balance have been suggested in the pathogenesis of psoriasis (Bovenschen et al., 2011; Soler et al., 2013; Zhang et al., 2016). It has been demonstrated that GPX4 is significant in protecting Treg cells from undergoing lipid peroxidation and ferroptosis, thereby contributing to maintaining immune homeostasis (Xu et al., 2021). GPX4 has an inhibitory effect on Th1 and Th17 responses at a steady state. Activated by T-cell receptors and co-stimulatory signals, *Gpx4*-knockout Treg cells exhibited abnormal accumulation of lipid peroxides and underwent ferroptosis. The deletion of *Gpx4* in Treg cells damaged the mitochondrial homeostasis and increased IL-1β generation, which in turn facilitated inflammatory responses. These results emphasize that GPX4 is required in Treg cells in order to prevent ferroptosis to maintain normal Treg cell function and sustain immune homeostasis (Xu et al., 2021).

Previous research results supported that activation of GPX4 contributes to the suppression of inflammation (Brigelius-Flohe, 2006; Meng et al., 2015). Research showed that activation of GPX4 was able to suppress inflammation, including cellular ROS level reduction and NF-κB pathway suppression, and suggested boosting GPX4 activity could be considered a novel anti-inflammatory strategy in lipid peroxidation-associated diseases (Li et al., 2018). There were differences in gene expression profiles in the skin samples of psoriasis patients and healthy individuals (Arbiser et al., 2018). Specifically, the expression level of GPX4 was reduced and the expression of *Nrf2* downstream target genes was increased in psoriasis lesions by comparison with healthy and uninvolved skin samples (Arbiser et al., 2018). Keratinocytes were proven to be sensitive to ferroptosis and the expression of multiple pro-inflammatory cytokines is upregulated. Treatment with Ferrostatin-1 effectively prevented erastin-induced ferroptosis in keratinocytes as well as reduced the level of pro-inflammatory cytokines. Mitochondria of keratinocytes were observed with classic ferroptotic morphological alterations in the skin lesions of the Imiquimod-induced psoriasis-like mice. Topical application of Ferrostatin-1 effectively reduced the severity of psoriasis-like lesions by decreasing the levels of various pro-inflammatory cytokines. Additionally, neither topical application of erastin nor RSL-3 exacerbated the severity of skin lesions in psoriasis-like mice, suggesting that ferroptosis is not an initiating factor for inducing psoriasis-like dermatitis, but rather emerges during the course of the disease and worsens cutaneous inflammation (Shou et al., 2021).

UV radiation (UVR) exposure can activate the cutaneous immune system by releasing pro-inflammatory factors, ROS, and inducing apoptosis in keratinocytes, thereby causing sterile inflammation or cancer (D'Orazio et al., 2013). Several studies have confirmed that UVR exposure can activate multiple PCD pathways in keratinocytes. Simultaneously, keratinocytes can maintain the homeostasis of the skin system by regulating different PCD pathways, including apoptosis, pyroptosis, necroptosis, and autophagy, to cope with UVR -related damage (Tang et al., 2021).

Vats et al. (2021) proved that UVR stimulation elicits ferroptosis in keratinocytes through various models. Keratinocytes exhibit typical ferroptosis alterations after UVB exposure, such as the accumulation of ROS and lipid peroxides. Also, different kinds of ferroptosis inhibitors, such as Ferrostatin-1 and liproxstatin-1, suppress UVB stimulation-induced keratinocyte ferroptotic cell death. Ferrostatin-1 could notably reduce the expression level of pro-inflammatory cytokines in the skin caused by UVB radiation and alleviate the infiltration level of immune cells in the dermis of the irradiated mouse. The sensitivity of keratinocytes to ferroptosis after UVB radiation is dependent on the levels of lipid peroxidation and GSH. Using metabolomic analysis, the discrepancies observed between the lipid profiles of keratinocytes after UVB exposure were found to originate mainly from oxidized PE, and the accumulation of the oxidized PE is believed to be essential for executing keratinocyte ferroptotic cell death after UVB radiation. Ferrostatin-1 potently inhibits the release of high mobility group box protein (HMGB)-1, a classical marker of necroinflammatory response, in cell models and human skin explants after UVB irradiation, thus confirming that ferroptosis is a key element of the necroinflammatory process in keratinocytes.

**FIGURE 6:**
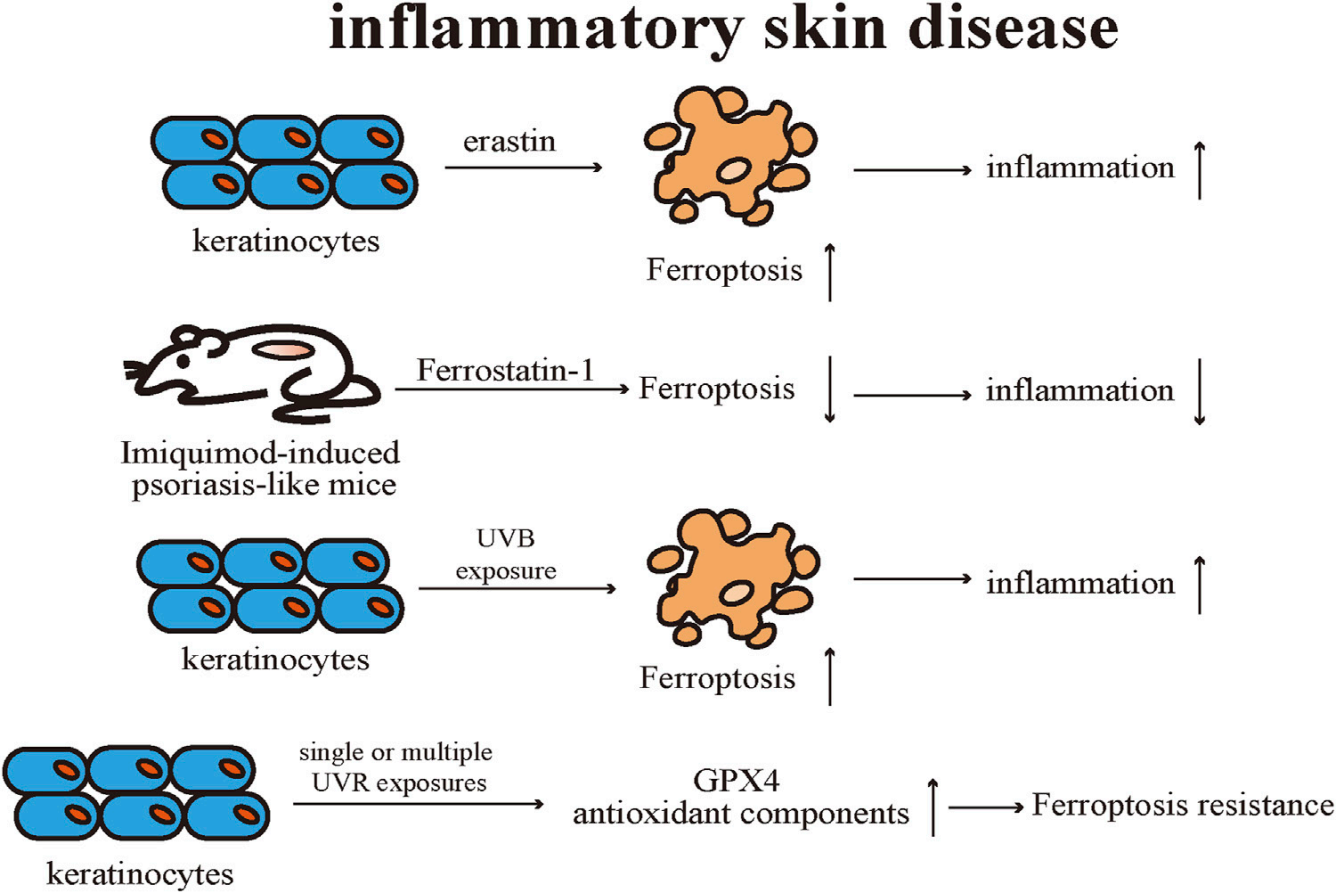
Keratinocytes are sensitive to ferroptosis, and ferroptosis inducers promote the secretion of proinflammatory cytokines. Topical application of ferroptosis inhibitors is effective in treating Imiquimod-induced psoriasis-like mice. UVB irradiation induces ferroptosis in keratinocytes, and UV radiation upregulates the levels of GPX4 and related antioxidant components resulting in ferroptosis resistance. Gpx4 exhibits limited protection against UV-induced ferroptosis in keratinocytes. GPX4: Glutathione peroxidases 4.

Feng et al. discovered that lipid peroxide accumulation and abnormal iron metabolism can occur in keratinocytes after single or multiple UVR exposures. Meanwhile, the expression levels of GPX4 and related antioxidant components were up-regulated after multiple radiation exposures, and thus keratinocytes manifested limited resistance to ferroptosis. However, such limited resistance to ferroptosis is forfeited under conditions that ferric ammonium citrate is introduced to mimic intracellular iron overload. Therefore, the limited protective effect of GPX4 against keratinocyte ferroptotic cell death could be associated with the disruption of the GPX4 function by oxidative damage after multiple UVB radiation. Nicotinamide mononucleotide (NMN), a precursor of NAD+, effectively restores intracellular redox imbalance induced by UVB radiation in keratinocytes. NMN upregulated the expression level of GPX4 in UVB-irradiated and iron overload-stimulated keratinocytes and alleviated the mice’s skin injury after UVB radiation. But, NMN is unable to function as described above in GPX4-knockdown keratinocytes, suggesting that the mechanism of ferroptosis resistance exerted by NMN is still reliant on GPX4 (Feng et al., 2022) (Figure 6).

## Autoimmune disease

Systemic Lupus Erythematosus (SLE) is a classic autoimmune disease with characteristic skin lesions, and the skin is the second most commonly involved organ in SLE patients (Obermoser et al., 2010; Ribero et al., 2017). SLE can induce immune dysfunction which includes the production of autoantibodies and defective clearance of immune complexes, leading to multi-organ damage (Fava and Petri, 2019). Li et al. (2021) discovered that peripheral blood neutrophils from patients with active SLE had elevated levels of intracellular lipid peroxides and exhibited the typical morphological changes of ferroptotic cell death. Peripheral blood neutrophil death in patients and SLE-prone mice could be inhibited by classical ferroptosis inhibitors, liproxstatin-1, and deferoxamine, while necroptosis and apoptosis inhibitors had relatively less effect on neutrophils, confirming that ferroptosis is the predominant neutrophil death pathway in SLE patients. Elevated serum levels of autoantibodies and IFN-α in SLE patients trigger ferroptosis by downregulating GPX4 expression while increasing intracellular lipid peroxidation levels in neutrophils. Meanwhile, myeloid-cell specific GPX4 haploinsufficient mice presented with SLE-like manifestations such as spontaneous skin lesions and systemic damage, further validating that ferroptosis of neutrophils serves a critical role in the pathogenies of SLE.

**FIGURE 7:**
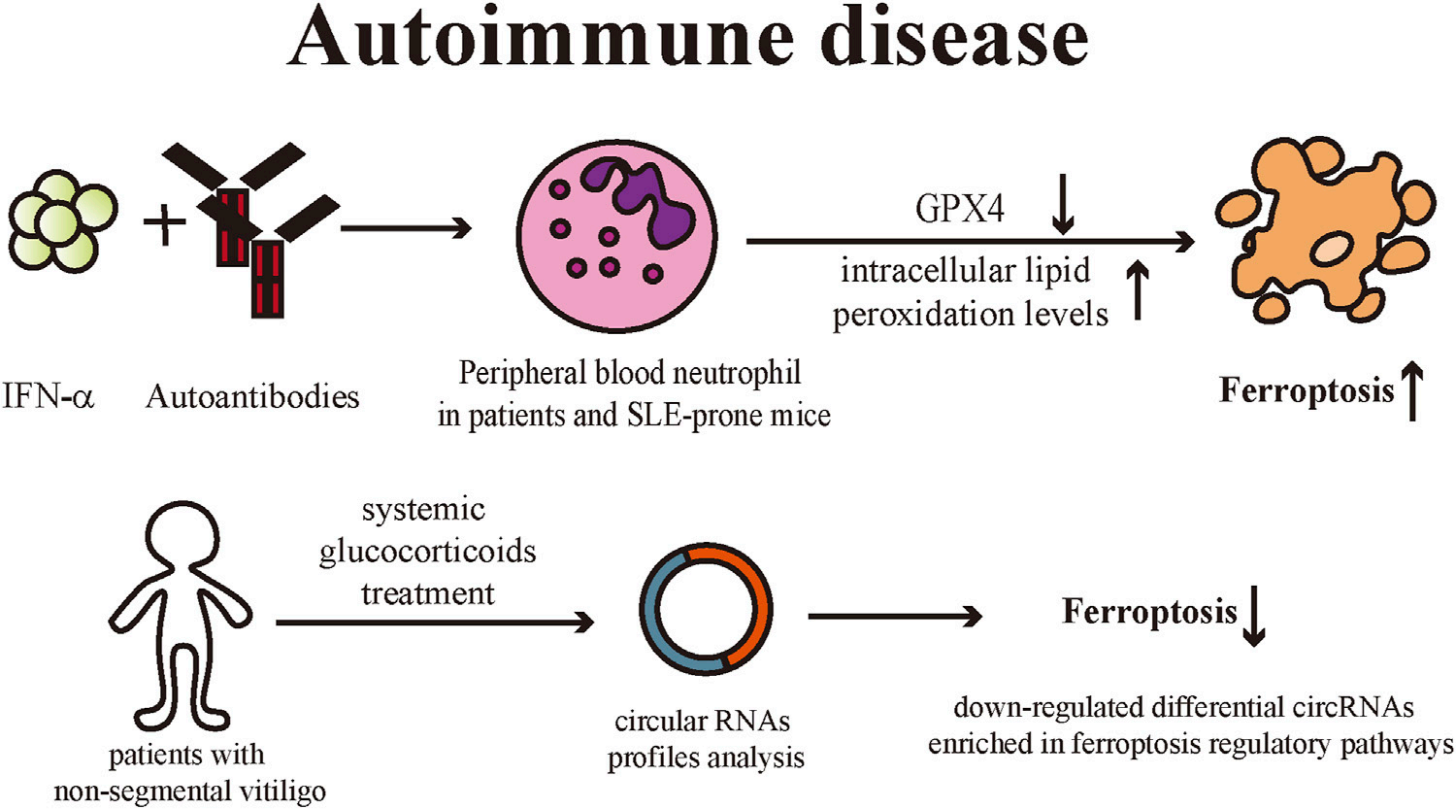
IFN-α and autoantibodies downregulate the GPX4 levels in peripheral blood neutrophils of SLE patients or SLE-prone mice, leading to increased intracellular lipid peroxidation levels and eventually inducing ferroptosis. Non-segmental vitiligo patients treated with systemic glucocorticoids exhibited down-regulation of ferroptosis-related circRNA expression profile in peripheral blood, suggesting that ferroptosis is involved in vitiligo pathogenesis. IFN: interferon; GPX4: Glutathione peroxidases 4; SLE: Systemic Lupus Erythematosus; circRNAs: circular RNAs.

Vitiligo is a commonly occurring skin depigmentation disease and is now recognized as an autoimmune disease (Frisoli et al., 2020). Multiple factors are involved in the pathogenesis of vitiligo, including genetics, innate and adaptive immune dysregulation, and oxidative stress. Raised ROS level is a critical contributor to the initiation and development of vitiligo pathophysiological processes (Bergqvist and Ezzedine, 2021). The generation of oxidative stress in keratinocytes and melanocytes has been demonstrated to be associated with the development of vitiligo. Melanocytes of vitiligo patients are more susceptible to oxidative stress (Puri et al., 1987). Under the stimulation of oxidative stress, keratinocytes are induced to produce CXC chemokine ligand (CXCL) 16, which further recruits CXCR6^+^ CD8^+^T cells and the infiltration of such T cells in the skin is followed by the loss of melanocytes in vitiligo lesions (Li et al., 2017). Zhang et al. (2022) analyzed circular RNAs (circRNA) profiles in peripheral blood samples before and after systemic treatment with glucocorticoid in patients with non-segmental vitiligo. CircRNA expression profiles of vitiligo patients before and after systemic glucocorticoids treatment were significantly different, and the Kyoto Encyclopedia of Genes and Genomes (KEGG) pathways revealed that the down-regulated differential circRNAs were predominantly enriched in ferroptosis regulatory pathways. Therefore, they suggested that the ferroptosis process was inhibited in vitiligo patients after systemic treatment. Although this discovery has not been verified by wet-lab biochemical experiments, they provide novel perspectives for the investigation of vitiligo (Figure 7).

## Conclusion and perspectives

As a significant kind of PCD, ferroptosis can be triggered pharmacologically or genetically. The depletion of GPX4 and GSH, the increased level of lipid peroxidation, and disrupted iron metabolism are vital features of ferroptosis (Dixon et al., 2012). Over the last few years, substantial progress has been made toward understanding the molecular and metabolic mechanisms of ferroptosis. It has been demonstrated that lipid peroxidation and subsequent ferroptosis following abnormal ALOX gene expression in keratinocytes are involved in the normal epidermal differentiation and maintenance of skin homeostasis, and might also engage in the development of skin squamous cell carcinoma (Egolf et al., 2021). However, it is currently inconclusive what specific roles ferroptosis exerts during the development of the skin and appendages, as well as in the pathophysiological processes of cutaneous diseases.

Being a novel mode of cell death, ferroptosis is known to be related to tumor progression and therapeutic efficacy. Ferroptosis provides a new perspective to elucidate how oncogenic and tumor-suppressive changes act in tumor progression. The relationship between malignant melanoma and ferroptosis has aroused extensive interest among scientists worldwide. Treatments for malignant melanoma remain challenging for dermatologists, such as drug resistance and non-response to treatments (Hargadon, 2021). It is urgent to identify effective combination therapeutic regimens that can better optimize the use of ferroptosis in the treatment of cancers. Activating ferroptosis could be an effective solution to treat certain kinds of tumors and overcome drug resistance to tumor treatments. Deepening the insight into ferroptosis is obviously more conducive to treating the associated disorders.

The phospholipids of the cell membrane maintain the integrity as well as the homeostasis of cells. However, the initiation of ferroptosis might compromise the structural integrity of the defense barriers and contribute to the invasion of microorganisms. Based on current investigations, ferroptosis triggered by *P. aeruginosa* and *M. tuberculosis* is confined to specific cells, whether the skin diseases they cause are related to ferroptosis still requires further research (Amaral et al., 2019; Dar et al., 2018).

There are strong pieces of evidence suggesting that ferroptosis exacerbates inflammation through immunogenicity, and several ferroptosis inhibitors have exhibited anti-inflammatory capabilities in various laboratory models of diseases (Kim et al., 2019). However, the point cannot be ignored that ferroptosis can protect cells from inflammatory damage in specific situations. Hence, it is vital to correctly determine whether ferroptosis exerts an anti- or pro-inflammatory effect on inflammation. Since the role exerted by ferroptosis in inflammation is not definite, under what conditions does ferroptosis have anti- or pro-inflammatory effects? Meanwhile, we have to evaluate the impact of ferroptosis on the immune system in both the short and long term.

On top of this, there are questions regarding ferroptosis that should be further deliberated as well. Many shreds of evidence support that there may be crosstalk among different cell death phenotypes. Critical modulators of ferroptosis exhibit regulatory capability in various PCD pathways. GPX4 responds to various tissue injuries by inhibiting different cell death pathways, such as apoptosis, pyroptosis, and necroptosis (Canli et al., 2016; Kang et al., 2018; Ran et al., 2004). The activation of autophagy may also induce alterations in ferritin, increasing the intracellular level of iron and subsequently promoting ferroptosis. So, how are the various types of cell death related to each other? Whether these types of cell death pathways can integrate to form a comprehensive modulatory network? Meanwhile, distinguishing between different kinds of cell death is still challenging. There remains an urgency to discover markers that can specifically separate ferroptosis cells from other kinds of PCDs, as well as its downstream consequences. Further elucidation of such interrelationships is also necessary for exploring the underlying mechanisms and developing novel therapeutic options.

Overall, the identification of ferroptosis offers novel directions for the research of cutaneous diseases. Ferroptosis has a critical role in the pathogenesis of various cutaneous diseases. However, the regulatory mechanism of ferroptosis has not been fully elucidated and the investigation still faces challenges. Gaining a deeper insight into what role ferroptosis play in cutaneous diseases could help provide and optimize therapeutic options.
